# Preanalytical conditions of point-of-care testing in the intensive care unit are decisive for analysis reliability

**DOI:** 10.1186/s13613-016-0152-6

**Published:** 2016-06-24

**Authors:** Adrien Auvet, Fabien Espitalier, Leslie Grammatico-Guillon, Mai-Anh Nay, Djilali Elaroussi, Marc Laffon, Christian R. Andres, Annick Legras, Stephan Ehrmann, Pierre-François Dequin, Chantal Gendrot, Antoine Guillon

**Affiliations:** Service de Réanimation Polyvalente, CHRU Tours, 2 Boulevard Tonnellé, 37000 Tours, France; Département d’Anesthésie et Réanimation, CHRU Tours, 2 Boulevard Tonnellé, 37000 Tours, France; Faculté de Médecine, Université François Rabelais, 10 Boulevard Tonnellé, 37032 Tours Cedex, France; Service d’Information Médicale, épidémiologie et économie de la santé, Hôpital Bretonneau, CHRU Tours, 2 Boulevard Tonnellé, 37000 Tours, France; Laboratoire de Biochimie, CHRU Tours, 2 Boulevard Tonnellé, 37000 Tours, France; Centre d’Etude des Pathologies Respiratoires, U1100, INSERM, 10 Bd Tonnellé, 37032 Tours, France

**Keywords:** Point-of-care testing, Preanalytical, Intensive care, Electrolyte, Hemoglobin

## Abstract

**Background:**

Point-of-care testing (POCT) systems enable a wide range of tests to be rapidly performed at the bedside and have attracted increasing interest in the intensive care unit (ICU). However, previous studies comparing the concordance of POCT with central laboratory testing have reported divergent findings. Most reported studies on POCT reliability have focused on analyzer performance rather than the preanalytical phase. The aim of this study was to assess the reliability of results provided by point-of-care analyzers according to the organization of the care units and the preanalytical process.

**Methods:**

In three adult critical care units, 491 paired blood samples were analyzed for hemoglobin, potassium, and sodium concentrations by blood gas analyzers (identical reference) and the central laboratory. The clinical significance of agreement was assessed using Bland–Altman plots. A quality improvement program was then implemented to improve the preanalytical POCT process for one ICU where there was poor agreement. A second comparison was performed on 278 paired blood samples in this unit.

**Results:**

Biases were clinically nonsignificant for potassium and sodium concentrations for all tested critical care units, relative to the reference method. However, biases [limits of agreements] for hemoglobin analyses were clearly affected by the preanalytical process: −3 [−6; 1] g/L in the operating room, −5 [−28; 17] g/L in a 10-bed ICU, and −19 [−64; 27] g/L in a 37-bed ICU. The quality approach was implemented in the 37-bed ICU and led to corrective actions that: (1) reduced the time for the POCT preanalytical phase; (2) implemented a checklist to validate the preanalytical conditions; (3) used technical innovations. The improvement of the preanalytical process resulted in a substantial decrease of the bias for hemoglobin concentration measurements: −3 [−10; 5] g/L in the 37-bed ICU.

**Conclusion:**

We clearly demonstrate that an identical analyzer can provide results of varying quality depending on the local constraints of the ICUs. We demonstrate that quality management focused on the preanalytical process and performed by the partners involved in the POCT can overcome these issues.

## Background

Point-of-care testing (POCT) can be defined as a rapid biological test near the site of care of a patient or at the bedside [1]. One of the major advantages of POCT is the reduction of the laboratory test turnaround time to rapidly treat the patient as appropriate [2, 3]. POCT systems have been developed over the past few years and have enabled a wide range of tests to be rapidly and simply performed without using sophisticated central laboratory equipment. Intensive care units (ICUs) have thus increasingly used POCT as a routine element of patient management [4–7], notably for blood gas analysis [8]. The primary objective of a blood gas analyzer (BGA) is to deliver accurate measures of blood gas levels (pH, *p*O_2_, *p*CO_2_); however, modern analyzers can currently measure additional parameters in the same blood sample, such as hemoglobin or electrolyte concentrations (sodium, potassium, chloride, and ionized calcium). POCT provides an adequate solution to obtain immediate results at the bedside [4], as abnormal blood electrolyte or hemoglobin concentrations need to be promptly corrected for ICU patients. Nevertheless, previous studies assessing the concordance of POCT with central laboratory testing have reported divergent findings. Overdiagnosis of anemia or significant differences in electrolyte concentrations relative to central laboratory analyzers have been reported for POCT analyzers [9–11], whereas interchangeability was observed in others [6, 9, 10, 12]. Given the importance of laboratory test results on the final medical decision-making process, laboratory analysis errors largely contribute to the overall risk of error. Accordingly, a majority of intensivists do not base important clinical decisions on POCT results. Only 48 % of clinicians make clinical decisions based on the potassium results obtained using a BGA without waiting for confirmation from the central laboratory [13, 14]. POCT in the ICU is currently used as complement to conventional laboratory services for the analysis of electrolyte and hemoglobin, but it is not a substitute, clearly limiting its impact and raising doubts about its relevance [8].

The increasing use of POCT is particularly challenging for POCT suppliers. POCT analyses are performed by non-laboratory staff, in diverse clinical contexts, in which the POCT users have varying levels of experience with the device. Suppliers of POCT analyzers are consequently concerned about ensuring consistency and quality of the analyses. The preanalytical phase, performed outside the direct supervision of laboratory professionals, could be an important reason for the underperformance of POCT [8]. However, most reported studies on POCT reliability have been mainly focused on the performance of the analyzer [9–11, 15] and not the preanalytical phase, including blood sampling and handling. Laboratory medicine is, not surprisingly, subject to error, but it is less well known that most laboratory errors occur during the manually intensive activities of the preanalytical phase [16–18]. The aim of this study was to assess the reliability of POCT analyses in various testing environments and specific clinical contexts. We first studied the results for hemoglobin, potassium, and sodium concentration measurements provided by BGAs delocalized in three specific clinical departments and compared these to the central laboratory results. The preanalytical management was modified due to substantial analytical errors observed in different situations. We finally evaluated the impact of the preanalytical modifications on the POCT performance.

## Methods

### Setting

Three critical care units were involved in this study (see Table 1 for a detailed description):

- Cardiac Surgery Operating Room (OR) with a BGA dedicated to one patient,
- Neurosurgical ICU (10-bed ICU) with moderate BGA activity,
- Polyvalent ICU (37-bed ICU) with elevated BGA activity.

**Table 1:** Detailed features of each care unit

	Care unit		
	Cardiac Surgery OR	Neurosurgical ICU	Polyvalent ICU
Number of beds in the critical care unit	1	10	37
Nurse-to-patient ratio	1:1	1:2.5	1:3.7
Daily mean number of POCT analysis	20	7	55
BGA location	Adjacent room in the cardiac surgery area, at a distance of 5 m	Central in the ICU, maximum of 32 m from the farthest room	Central in the ICU, maximum of 80 m from the farthest room
Blood collection by arterial cannulation	Yes	Yes	Yes
Care-giver in charge of the blood sampling	Anesthesiologist and/or nurse anesthetist	Nurse	Nurse
Care-giver in charge of the POCT analysis	Anesthesiologist and/or nurse anesthetist	Nurse	A care team member devoted to all POCT analyses (per quarter)
POCT user education	Initial education, regular information	2 h a year, regular information	2 h quarterly, at traineeship start, and regular information
Phone assistance for POCT (24/7)	Yes	Yes	Yes
Hemoglobin measurement on BGA	Yes	Yes	Yes
Potassium and sodium concentration measurement on BGA	Yes	No	Yes
Blood gas syringe used for POCT	SafePICO^®^ syringe	SafePICO^®^ syringe	Syringe BD Preset™

Characteristics of point-of-care testing organization for each care unit

*OR* operative room, *ICU* intensive care unit, *POCT* point-of-care testing, *BGA* blood gas analyzer

### Blood sampling and analysis

All patients included in the study had arterial cannulation for ongoing blood pressure monitoring, which also allowed the simultaneous collection of blood samples from the arterial cannula for both blood gas POCT and central laboratory analysis. Purging of the arterial perfusion line was performed using a standard (5 mL) closed system (Blood management system VAMP™ 60 in with Arm-mount Reservoir, Edwards Lifesciences™, Irvine, USA). A portion of the blood specimen was placed directly into a heparinized syringe for blood gas analysis and into collection tubes for central laboratory analysis (BD Vacutainer^®^, Le Pont de Claix, France). Paired samples were generated for each patient. The blood samples were thereafter transported at room temperature to the BGA and to the central laboratory for analysis.

All POCT for blood gas analysis was performed using the ABL 825^®^ FLEX analyzer (Radiometer, Copenhagen, Denmark). Sodium and potassium measurements were based on the potentiometric method, performed by an ion-selective electrode. The total hemoglobin concentration was measured by spectrophotometry. The syringes used were: Syringe BD Preset™ (BD, Franklin Lakes, NJ, USA) in the 37-bed ICU and SafePICO^®^ syringe (Radiometer, Copenhagen, Denmark) in the Cardiac Surgery OR and the 10-bed ICU. All BGA analyses were performed according to the manufacturers’ guidelines with special attention to the preanalytical conditions, including prevention of air contamination in the syringe, immediate analysis after collection, and proper mixing of blood syringes before introduction in the analyzer. Quality assurance according to the laboratory’s quality control procedure was performed on the BGAs and central laboratory analyzers. The maintenance of the BGAs was performed by the biochemical staff in accordance with the manufacturer’s guidelines. The results for automatic quality controls and calibrations were verified by the same biological staff.

In the central hospital laboratory, the hemoglobin concentration was analyzed using a Beckman Coulter Unicel^®^ DxH 800 analyzer (Beckman Coulter Inc., Miami, FL, USA) and electrolytes using an AU2700 chemistry analyzer (Beckman Coulter Inc., Miami, FL, USA). All analyses were performed by experienced laboratory technicians according to the manufacturer’s procedures. All instruments involved in the study were regularly maintained according to the manufacturer’s technical manual.

### Design

#### Step 1

We first calculated the biases between paired measurements of potassium, sodium, and/or hemoglobin blood concentrations between the POCT and central laboratory for each of the three departments. From the point of view of POCT, the Cardiac Surgery OR may be considered to be a model of a “10-bed ICU” with almost perfect preanalytical conditions; limited preanalytical variation was therefore expected. We assumed that the results obtained under these conditions could serve as reference for optimal preanalytical conditions (gold standard). We thus compared the biases obtained in the Cardiac Surgery OR to those obtained in 10-bed specialized ICU and 37-bed polyvalent ICU.

#### Step 2

A quality approach was implemented to improve the preanalytical process of the 37-bed ICU because large differences were observed between the biases calculated in the Cardiac Surgery OR and the 37-bed ICU (see “Results” section). Corrective actions were implemented after a multidisciplinary brainstorming involving the nurse managers, intensivists, and a clinical chemistry specialist. A new assessment of the POCT in the 37-bed ICU was then performed and compared to the gold standard.

### Ethical considerations

This prospective, observational, non-interventional study was conducted at the University Hospital of Tours (France). The treatment of personal health data of this observational research has been approved by the Commission Nationale de l’Informatique et des Libertés (CNIL). The study was also approved by an independent Research Ethics Committee (ERERC, Espace de Réflexion Ethique de la Région Centre). All patients included in this study were personally informed by a written document about the treatment of the data, as well as their right to object to the study and obtain access to the data, according to articles L.1121-1 and R1121-2 of the French Public Health Code. The need for individual patient consent was waived by the Research Ethics Committee as the study was considered to be a quality assurance project.

### Statistical analysis

Quantitative data were reported as median values and interquartile range (IQR). Comparisons of quantitative values were analyzed using the paired *t* test (for delay comparison, Gaussian distribution), and the Mann–Whitney or Kruskal–Wallis test (and Dunn’s test for post hoc comparisons) depending on the number of groups to analyze. Determination of the agreement between laboratory (reference method) and POCT values was performed as described by Bland and Altman [19]. We plotted the average of each paired analysis against the difference for the same pair (central laboratory value − POCT value). Results are presented as bias [inferior limit of agreement (−1.96 SD); superior limit of agreement (+1.96 SD)]. It has been previously reported that for the comparison of measurement methods, at least 53 paired-values for hemoglobin were necessary in operating theaters [20], whereas 127–202 consecutive paired analyses were necessary for comparison in ICUs where higher variability is expected [21, 22]. Statistical analyses were performed using GraphPad Prism^®^ v.5.0, and a *p* value <0.05 was considered to be significant.

## Results

### Step 1

In the first step of the study, we evaluated whether the measurement of hemoglobin and electrolyte (potassium, sodium) concentrations using a BGA could provide a valid alternative method to conventional laboratory testing. Detailed results are listed in Table 2. The median time to obtain results was substantially reduced by POCT relative to the central laboratory in all critical care units:

- 2 [2–2] min instead of 82 [58–130] min (*p* < 0.0001) in the Cardiac Surgery OR,
- 5 [4–7] min instead of 80 [63–108] min (*p* < 0.0001) in the 10-bed ICU,
- 24 [13–38] min instead of 112 [90–135] min in the 37-bed ICU (*p* < 0.0001).

**Table 2:** Comparison of hemoglobin, potassium, and sodium concentration measurements provided by the BGA in three critical care units with different preanalytical processes

	Cardiac Surgery OR *n* = 53	10-bed ICU *n* = 178	37-bed ICU/Step 1 *n* = 260	37-bed ICU/Step 2 *n* = 278
Time for results (min)				
POCT	2 [2; 2]	5 [4; 7]*	24 [13; 38]*	15 [5; 29]*
Central lab.	82 [58; 130]	80 [63; 108]	112 [90; 135]	119 [80; 144]
Hemoglobin conc. (g/L)				
POCT	129 [120–137]	108 [99–121]	115 [96–138]	93 [81–116]
Central lab.	126 [116–134]	104 [95–116]	101 [82–112]	91 [79–110]
Bias [limits of agreement]	−3 [−6; 1]	−5 [−28; 17]	−19 [−64; 27]*	− 3 [−10; 5]
Potassium conc. (mmol/L)				
POCT	4.1 [3.9–4.3]	na	4 [3.5–4.3]	4.2 [3.8–4.7]
Central lab.	4.2 [4–4.5]	na	4.1 [3.7–4.4]	4.3 [3.9–4.8]
Bias [limits of agreement]	0.1 [−0.1; 0.4]		0.1 [−0.1; 0.4]	0.1 [−0,3; 0,5]
Sodium conc. (mmol/L)				
POCT	139 [137–140]	na	138 [135–142]	136 [134–139]
Central lab.	139 [137–140]	na	138 [135–142]	138 [135–140]
Bias [limits of agreement]	0 [−3; 3]		0 [−3; 3]	1 [−2; 4]

Results are expressed as the median [IQR] or bias [inferior limits of agreement; superior limits of agreement]. *n* refers to the number of paired analyses

*OR* operative room, *ICU* intensive care unit, *POCT* point-of-care testing, *lab.* laboratory, *conc.* concentration, *med.* median, *na* non-available

* *p* < 0.001 (Cardiac Surgery OR vs. 10- or 37-bed ICU)

The time required to obtain the results from the central laboratory did not significantly differ depending on the care units. In contrast, the turnaround times for POCT analyses were significantly different and markedly longer in both the 10-bed and 37-bed ICUs relative to the Cardiac Surgery OR (*p* < 0.0001 for 10- or 37-bed ICU versus Cardiac Surgery OR and *p* < 0.0001 for 10- versus 37-bed ICU).

**Fig. 1:**
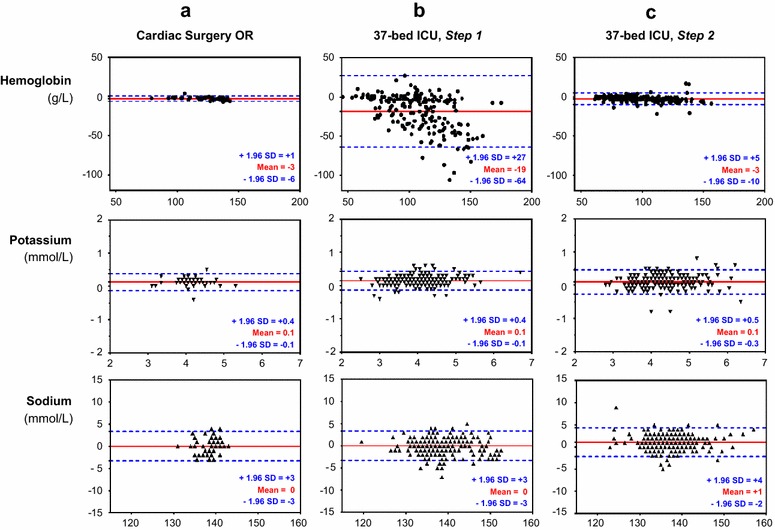
Bland–Altman plots for the comparisons between each method for estimating the hemoglobin, potassium, and sodium concentrations. Comparisons of the analyses were performed in **a** cardiac surgery operative room (OR), **b** polyvalent intensive care unit of 37 beds (37-bed ICU). The preanalytical process of the 37-bed ICU was improved according to the multidisciplinary quality approach, and a second run of comparisons was performed in this critical care unit (**c**). The *solid red line* in each Bland–Altman plot indicates the mean difference (bias) between the methods (value from the central laboratory − value from blood gas analyzer delocalized in the intensive care unit); the *broken blue lines* indicate the 95 % limits of agreement

The bias and limits of agreements for hemoglobin concentrations in the Cardiac Surgery OR, 10- and 37-bed ICUs were: −3 [−6; 1], −5 [−28; 17], and −19 [−64; 27] g/L, respectively (Fig. 1a, b). The biases for hemoglobin concentration were significantly different between the 37-bed ICU and the Cardiac Surgery OR (*p* < 0.0001), whereas we observed no statistical difference between the 10-bed ICU and Cardiac Surgery OR (*p* = 0.14).

The Bland–Altman plots for potassium and sodium concentrations in the Cardiac Surgery OR or 37-bed ICU are shown in Fig. 1a, b. The biases and limits of agreements for the electrolyte measurements were not significantly different between the Cardiac Surgery OR and the 37-bed ICU (0 [−0.9; 0.8] and 0.1 [−0.1; 0.4] mmol/L for potassium in the Cardiac Surgery OR and the 37-bed ICU, respectively [*p* = 0.17]; 0 [−3; 3] and 0 [−3; 3] for sodium in the Cardiac Surgery OR and the 37-bed ICU, respectively [*p* = 0.81]).

### Step 2

A quality approach was implemented due to the poor agreement between the reliability of results provided by the BGA of the 37-bed ICU relative to the gold standard (BGA of Cardiac Surgery OR). The corrective actions were as follows:

- All morning blood tests were routinely scheduled for analysis at 7:00 a.m. and a rush hour due to multiple analyses resulted in an increase of the POCT turnaround time. We therefore reorganized the scheduled morning analysis to extend from 4:00 to 7:00 a.m. in three periods.
- We implemented a checklist to validate the preanalytical conditions and set the maximum time allowed for the preanalytical phase.
- We added a module on the ABL825^®^ FLEX analyzer to provide automatic sample handling by successively identifying, mixing, aspirating, and measuring up to three blood gas samples (FLEXQ^®^, Radiometer, Copenhagen, Denmark). Previous studies have clearly demonstrated the superiority of automatic mixing to produce a homogeneous sample [23, 24]. Moreover, this device reduces the turnaround time of the analyses due to an automatic syringe change.
- We needed to switch the BD Preset™ 1-mL (BD, Franklin Lakes, NJ, USA) syringe for the SafePICO^®^ syringe (Radiometer, Copenhagen, Denmark) to use the automatic FLEXQ^®^ device.

We assessed the impact of the implementation on the process after modification of the preanalytical process of the 37-bed ICU based on the multidisciplinary quality approach. We observed a shorter POCT turnaround time relative to the first investigation: The duration of 24 [13–38] min in Step 1 was reduced to 15 [5–29] min in Step *2* (*p* < 0.0001, Table 2). The time to obtain results for the central laboratory was unchanged (112 [90–135] min in Step 1 vs. 119 [80–144] min in Step 2, *p* = 0.85).

The bias for hemoglobin concentration was drastically reduced and was significantly different from that measured in Step 1: −2 [−10; 5] g/L instead of −19 [−64; 27] g/L (*p* < 0.0001) (Fig. 1c). We did not observe a significant difference in the bias for the hemoglobin concentration between the 37-bed ICU (Step 2) and the Cardiac Surgery OR (*p* = 0.44). The biases for the potassium and sodium concentrations were not modified by the modified preanalytical conditions of Step 2: 0.1 [−0.2; 0.4] mmol/L for potassium and 1 [−2; 4] mmol/L for sodium.

## Discussion

POCT has obvious theoretical advantages in ICUs: Numerous variables in a limited blood sample can be rapidly measured at the bedside without wasting time by transporting or centrifuging the sample. However, some concerns have been raised about the reliability of electrolyte and hemoglobin measurements performed by BGAs in clinical departments. Many hypotheses have been put forward to explain the discrepancies between POCT and core laboratory analyzers. The assumptions were mainly focused on analyzer performance. To our knowledge, we have clearly demonstrated, for the first time, that the preanalytical phase is the principal limiting factor in the ICU to obtain sufficient agreement between POCT and laboratory analyzers. Finally, we highlight that preanalytical quality management, resulting from a multidisciplinary, point-by-point analysis, can lead to substantial improvement in the final results provided by POCT analyzers.

 We considered the preanalytical conditions in the Cardiac Surgery OR to be almost perfectly controlled and assumed that the results obtained could serve as a reference for the optimal preanalytical phase of an ideal “10-bed ICU.” According to the US Clinical Laboratory Improvement Amendment (USCLIA) [25], potassium, sodium, and hemoglobin biases found within ±0.5, ±4 mmol/L, and ±7 %, respectively, of the target value are acceptable. Indeed, the results obtained in 2 min by POCT in the Cardiac Surgery OR were perfectly acceptable: Potassium, sodium, and hemoglobin biases were 0.1 [−0.1; 0.4], 0 [−3; 3] mmol/L, and −3 [−6; 1] g/L, respectively (corresponding to 2.4 % of the target value). Thus, we confirmed that POCT of electrolytes and hemoglobin on a BGA (ABL 825^®^ FLEX analyzer) generates rapid and accurate biological results under optimal preanalytical conditions. We were able to extrapolate this conclusion from a “10-bed ICU” to a 10-bed ICU, but failed with a larger ICU of 37 beds. We demonstrated that the preanalytical conditions were the principal cause of the final analytical errors, as the analyzers were identical. Furthermore, all blood samples were collected in the same way on a dedicated arterial line that also excluded common errors of blood sampling, including hemolysis and sample contamination. It is thus highly probable that the large analytical discrepancies observed in the largest ICU were due to the difficulty of ensuring the consistency and quality of the preanalytical conditions. Indeed, it appears possible to control the source of errors in a 1- to 10-bed ICU, but it is clearly more challenging to minimize human error in a substantial ICU with a heavy workload.

The preanalytical phase of POCT could be particularly complex in the context of the ICU. It may be more difficult to standardize preanalytical processes than the analytical phase due to the various clinical situations. The preanalytical phase is probably different from one patient to another depending on the degree of emergency or severity. We developed a quality control strategy using a global, multidisciplinary approach, developed by representatives of clinical, laboratory, and nursing staff, to determine and remove the cause of these random errors [26]. The quality control strategy included all steps of the analysis and was set up to reflect the exact conditions of clinical practice. Indeed, we systematically examined this process, from the bedside to the analyzer, and defined a small number of individual steps that must imperatively be carried out. We finally proposed a new care bundle to reduce variability of the preanalytical phase of POCT in the 37-bed ICU: definition of the upper time limit for the preanalytical phase according to the existing guidelines and local constraints [17]; modification of the organization of the ICU to guarantee this limited delay; the use of existing technology to standardize the mixing of blood samples [23, 24]; implementation of a short checklist to confirm the successful completion of each step. With this care bundle approach, we emphasized the importance of pragmatically completing all important elements of care (rather than considering each element individually). Finally, we reduced the turnaround time between Steps 1 and 2 by 38 % (24 [13–38]–15 [5–29] min). The benefit of the care bundle was obvious in Step 2: The bias and unwarranted variation for hemoglobin was significantly decreased from −19 [−64; 27] to −2 [−10; 5] g/L.

This global approach may paradoxically be a limitation in our study. We are unable to identify the precise role of each modification because all the modifications occurred at the same time. For example, we did not specifically assess the impact of reducing turnaround time on the final success of this care bundle. The choice of the syringe for POCT is also a well-known pitfall [27] that we did not investigate here. However, the study goal was not to puzzle out the random errors of the preanalytical phase through a series of deductions and research. It seems inappropriate, and even unethical, to determine step by step the improvement of POCT. On the contrary, the strength of our strategy was in supporting a pragmatic analysis and then to tie the changes together into a bundle of interventions that was fully respected. Another limitation of the study design is the absence of sodium and potassium measurements in the 10-bed ICU. However, we observed that the measurement of these electrolyte concentrations were not sensitive to the various preanalytical phases.

The suppliers of POCT are fully aware of their role in providing user assistance to guarantee the correct professional use of their tests, therefore providing reliable and safe results [28, 29]. As POCT is performed outside the walls of the laboratory by staff with a limited technical background, training and quality control can be critical [30]. Internal quality programs are already implemented by POCT suppliers: For example, the BGA automatically performs quality controls, checks the analytical system, initiates necessary corrective actions, and documents all activities. However, the results provided by this study demonstrated that these systems cannot overcome the issues generated during the preanalytical phase. Finally, our results underline that the underperformance of POCT can be significantly improved to provide more reliable results by a tight collaboration between users (ICU staff) and providers (laboratory staff).

## Conclusion

Studies on the reliability of POCT of electrolytes and hemoglobin in the ICU by BGAs have provided conflicting results. However, these previous studies were mainly focused on the analyzer rather than the preanalytical phase. The strength of this study was in integrating the global process of POCT, from the bedside to the final result. To our knowledge, we clearly demonstrate, for the first time, that an identical analyzer can provide results of varying quality depending on the local constraint of the ICUs. Further, we demonstrate that quality management performed by the partners involved in the POCT can overcome these issues. It is now possible to bring laboratory tests closer to the patient, but it is imperative to monitor their reliability in the exact condition of utilization.

## Abbreviations

POCT: point-of-care testing
ICU: intensive care unit
BGA: blood gas analyzer
OR: operative room

## References

[1] Kost GJ, Ehrmeyer SS, Chernow B, Winkelman JW, Zaloga GP, Dellinger RP, Shirey T (1999). The laboratory-clinical interface: point-of-care testing. Chest.

[2] Lee EJ, Shin SD, Song KJ, Kim SC, Cho JS, Lee SC, Park JO, Cha WC (2011). A point-of-care chemistry test for reduction of turnaround and clinical decision time. Am J Emerg Med.

[3] Kendall J, Reeves B, Clancy M (1998). Point of care testing: randomised controlled trial of clinical outcome. BMJ.

[4] Casagranda I (2010). Point-of-care testing in critical care: the clinician’s point of view. Clin Chem Lab Med.

[5] Schwarzer P, Kuhn S-O, Stracke S, Gründling M, Knigge S, Selleng S, Helm M, Friesecke S, Abel P, Kallner A, Nauck M, Petersmann A (2015). Discrepant post filter ionized calcium concentrations by common blood gas analyzers in CRRT using regional citrate anticoagulation. Crit Care.

[6] Inoue S, Egi M, Kotani J, Morita K (2013). Accuracy of blood-glucose measurements using glucose meters and arterial blood gas analyzers in critically ill adult patients: systematic review. Crit Care.

[7] Rooney KD, Schilling UM (2014). Point-of-care testing in the overcrowded emergency department—can it make a difference?. Crit Care.

[8] Nichols JH, Christenson RH, Clarke W, Gronowski A, Hammett-Stabler CA, Jacobs E, Kazmierczak S, Lewandrowski K, Price C, Sacks DB, Sautter RL, Shipp G, Sokoll L, Watson ID, Winter W, Zucker ML, National Academy of Clinical Biochemistry (2007). Executive summary. The National Academy of Clinical Biochemistry Laboratory Medicine practice guideline: evidence-based practice for point-of-care testing. Clin Chim Acta Int J Clin Chem.

[9] King R, Campbell A (2000). Performance of the radiometer OSM3 and ABL505 blood gas analysers for determination of sodium, potassium and haemoglobin concentrations. Anaesthesia.

[10] Morimatsu H, Rocktäschel J, Bellomo R, Uchino S, Goldsmith D, Gutteridge G (2003). Comparison of point-of-care versus central laboratory measurement of electrolyte concentrations on calculations of the anion gap and the strong ion difference. Anesthesiology.

[11] Leino A, Kurvinen K (2011). Interchangeability of blood gas, electrolyte and metabolite results measured with point-of-care, blood gas and core laboratory analyzers. Clin Chem Lab Med.

[12] Zhang JB, Lin J, Zhao XD (2015). Analysis of bias in measurements of potassium, sodium and hemoglobin by an emergency department-based blood gas analyzer relative to hospital laboratory autoanalyzer results. PLoS One.

[13] José RJP, Preller J (2008). Near-patient testing of potassium levels using arterial blood gas analysers: can we trust these results?. Emerg Med J.

[14] Bloom BM, Connor H, Benton S, Harris T (2014). A comparison of measurements of sodium, potassium, haemoglobin and creatinine between an emergency department-based point-of-care machine and the hospital laboratory. Eur J Emerg Med Off J Eur Soc Emerg Med.

[15] Cembrowski GS, Tran DV, Higgins TN (2010). The use of serial patient blood gas, electrolyte and glucose results to derive biologic variation: a new tool to assess the acceptability of intensive care unit testing. Clin Chem Lab Med.

[16] Lippi G, Becan-McBride K, Behúlová D, Bowen RA, Church S, Delanghe J, Grankvist K, Kitchen S, Nybo M, Nauck M, Nikolac N, Palicka V, Plebani M, Sandberg S, Simundic A-M (2013). Preanalytical quality improvement: in quality we trust. Clin Chem Lab Med.

[17] Simundic A-M, Lippi G (2012). Preanalytical phase—a continuous challenge for laboratory professionals. Biochem Med.

[18] Lippi G, Banfi G, Church S, Cornes M, De CarliG, Grankvist K, Kristensen GB, Ibarz M, Panteghini M, Plebani M, Nybo M, Smellie S, Zaninotto M, Simundic A-M, European Federation for Clinical Chemistry and Laboratory Medicine Working Group for Preanalytical Phase (2015). Preanalytical quality improvement. In pursuit of harmony, on behalf of European Federation for Clinical Chemistry and Laboratory Medicine (EFLM) Working group for Preanalytical Phase (WG-PRE). Clin Chem Lab Med.

[19] Bland JM, Altman DG (1986). Statistical methods for assessing agreement between two methods of clinical measurement. Lancet.

[20] Giraud B, Frasca D, Debaene B, Mimoz O (2013). Comparison of haemoglobin measurement methods in the operating theatre. Br J Anaesth.

[21] Hinds LE, Brown CL, Clark SJ (2007). Point of care estimation of haemoglobin in neonates. Arch Dis Child Fetal Neonatal Ed.

[22] Ray JG, Post JR, Hamielec C (2002). Use of a rapid arterial blood gas analyzer to estimate blood hemoglobin concentration among critically ill adults. Crit Care.

[23] Grenache DG, Parker C (2007). Integrated and automatic mixing of whole blood: an evaluation of a novel blood gas analyzer. Clin Chim Acta Int J Clin Chem.

[24] Benoit M-O, Paul J-L (2009). Evaluation and advantages of an automatic magnetic mixing of syringes integrated to a whole blood gas analyser. Scand J Clin Lab Invest.

[25] Code of Federal Regulations. 42 CFR 493.941—hematology (including routine hematology and coagulation). 2010. http://www.gpo.gov/fdsys/granule/CFR-2010-title42-vol5/CFR-2010-title42-vol5-sec493-941 (Accessed 1 Oct 2010).

[26] Briggs C, Guthrie D, Hyde K, Mackie I, Parker N, Popek M, Porter N, Stephens C, British Committee for Standards in Haematology General Haematology Task Force (2008). Guidelines for point-of-care testing: haematology. Br J Haematol.

[27] van Berkel M, Scharnhorst V (2011). Electrolyte-balanced heparin in blood gas syringes can introduce a significant bias in the measurement of positively charged electrolytes. Clin Chem Lab Med.

[28] Briggs C, Kimber S, Green L (2012). Where are we at with point-of-care testing in haematology?. Br J Haematol.

[29] Legifrance. Ordonnance no. 2010-49 du 13 janvier 2010 relative à la biologie médicale. http://www.legifrance.gouv.fr/affichTexte.do?cidTexte=JORFTEXT000021683301&categorieLien=id (Accessed 15 Jan 2010).

[30] Price CP (2001). Point of care testing. BMJ.

